# Early amantadine treatment reduces the risk of death in patients with large hemisphere infarctions:a Chinese hospital-based study

**DOI:** 10.1186/s12883-021-02444-w

**Published:** 2021-10-28

**Authors:** Jie Li, Ping Zhang, Yingying Liu, Simiao Wu, Xingyang Yi, Shihong Zhang, Chun Wang, Ming Liu

**Affiliations:** 1 Department of Neurology, People’s Hospital of Deyang City, No.173, North Taishan Road, Deyang, 618000 Sichuan Province PR China; 2 Center of Cerebrovascular Diseases, Department of Neurology, West China Hospital, Sichuan University, No. 37 GuoXue Xiang, Chengdu, Sichuan Province 610041 PR China

**Keywords:** Amantadine hydrochloride, Large hemispheric infarction, Propensity score matching, Outcome

## Abstract

**Background:**

Amantadine hydrochloride is one of the most frequently prescribed drugs for patients with severe traumatic brain injury in restoring consciousness and accelerating the pace of functional recovery. However, there is a paucity of studies on the effectiveness of amantadine in patients with severe stroke especially large hemisphere infarction (LHI). The present study aimed to investigate whether amantadine treatment is associated with better clinical outcomes in conservatively treated LHI patients.

**Methods:**

We retrospectively collected conservatively treated LHI patients according to inclusion/exclusion criteria. The patients were divided into two groups based on the treatment regimen, whether they did receive amantadine hydrochloride in addition to standard therapy (ST) or not. The primary outcomes were in-hospital death, 3-month mortality, and unfavorable outcome (defined as modified Rankin Scale score of 4 to 6). All outcomes were compared between the two groups before and after propensity score matching (PSM). Multivariate logistic regression was performed to identify the association between early amantadine hydrochloride treatment and clinical outcomes in LHI patients.

**Results:**

Thirty-one LHI patients treated with amantadine combined with ST and 127 patients treated with ST were enrolled. Amantadine group had a shorter prehospital delay (median: 2 vs. 10 h), a higher baseline NIHSS score (21.71 ± 4.76 vs. 17.49 ± 5.84), and a higher rate of dominant hemisphere involvement (67.74% vs. 45.67%). After PSM, amantadine treatment significantly reduced the risk of in-hospital death (7.41% vs. 31.11%, *p*=0.019) and 3-month mortality (25.93% vs. 55.56%, *p*=0.008). Amantadine treatment yielded a significant decrease in death in-hospital (before PSM: OR 0.143, 95% CI 0.034 to 0.605; after PSM: OR 0.113, 95% CI 0.020 to 0.635) and 3-month mortality (before PSM: OR 0.214, 95% CI 0.077 to 0.598; after PSM: OR 0.176, 95% CI 0.053 to 0.586) in unmatched and matched multivariate analyses.

**Conclusion:**

The results of our study provide initial evidence that early amantadine treatment was associated with a decrease in death in conservatively treated LHI patients. Considering the limitations of observational study, randomized controlled trials with a large sample size may help provide a clearer picture of the utility of amantadine in LHI patients.

**Supplementary Information:**

The online version contains supplementary material available at 10.1186/s12883-021-02444-w.

## Background

Large hemispheric infarction (LHI), which usually results from occlusion of the internal carotid artery or proximal proximal middle cerebral artery (MCA), is one of the most devastating condition with high mortality and disability rate among acute ischemic stroke (AIS)patients [1–3]. Until recently, no pharmacological strategies have been proven effective by randomized controlled trials (RCTs) [ 4]. Although decompressive hemicraniectomy (DHC) within 48 h has been proven to benefit LHI patients with malignant brain edema (MBE) [5], only highly selected cases would be eligible for DHC based on the strict eligibility criteria in the DHC trials [6]. Furthermore, because of its invasive nature and the need for multidisciplinary cooperation, DHC is also underused worldwide. Although there is a lack of evidence, many neuropharmacological therapies are still used off-label due to the lack of effective treatment in conservatively treated LHI patients.

Amantadine was first synthesized more than 50 years ago and initially developed as an antiviral agent [7]. Further work has demonstrated the effectiveness of amantadine as an anti-parkinsonism agent [8, 9], as well as a treatment option for neuroleptic-induced extrapyramidal symptoms [10, 11] and neuroleptic malignant syndrome [12]. Amantadine hydrochloride is one of the most frequently prescribed drugs for patients with prolonged impaired consciousness after traumatic brain injury (TBI) [13]. The mechanism of action of amantadine is still not fully understood, but it is thought to act as an N-methyl-D-aspartate (NMDA) receptor antagonist increasing dopamine synthesis and release in the striatum [14]. Sufficient synaptic dopamine levels are necessary for many physiological functions including motivation, motor control, emotion, and cognitive processing [15]. The result of a small sample randomized trial in patients with TBI-associated diffuse axonal injury suggested that amantadine was effective in improving neuro-recovery and it was well tolerated at a dosage of 200 mg/day with no serious adverse side effects [16]. In a multicenter trial, amantadine at doses of 200–400 mg/day has successfully been demonstrated effective and safe in accelerating the pace of functional recovery in patients with severe TBI [17]. A clinical-experimental study conducted in Russian reported that amantadine exhibited significant restoration of consciousness and better regress of neurological deficit on the first day of AIS [18].

So far, there is a lack of studies investigating amantadine for the treatment of patients with non-traumatic brain injuries such as severe stroke. The present study aimed to explore whether amantadine treatment is associated with better clinical outcomes in conservatively treated LHI patients.

## Methods

### Study design and subjects

We performed a retrospective study using the prospective data of the Deyang Stroke Registry, which has been described previously [19, 20]. AIS patients who were admitted to People’s Hospital of Deyang City within 24 h from symptoms onset were consecutively registered from February 2012 to January 2015. LHI patients was defined as an infarction with computed tomography (CT) and/or magnetic resonance imaging (MRI) evidence of supratentorial cerebral infarction involving more than 50% of MCA region, with or without the involvement of the adjacent territories [21]. All patients completed a brain CT scan before initial treatment. A routine second CT or MRI scan was performed within the first 7 days of hospitalization. Other CT scans were performed in case of neurological deterioration occurred. Patients who met the following inclusion criteria were eligible for the present study: (1) age ≥ 18 years, (2) LHI patients admitted to hospital within 24 h of symptom onset. Patients were excluded if they met one of the following exclusion criteria: (1) received DHC during hospitalization; (2) prior treatment with amantadine; (3) history of severe renal disease; (4) history of epilepsy with more than one seizure in the previous month; (5) pregnancy; (6) incomplete hospital records or missing imaging that would prevent complete data collection; (7) any disability related to the central nervous system that predated the stroke; and (8) with a premorbid modified Rankin Scale (mRS) score of more than 2 and lived dependently [22].

All LHI patients were treated with standard therapy (ST), including mechanical ventilation in maintaining a patent airway, head position, glucose, blood pressure and temperature management, parenteral and enteral feeding, osmotic therapy for brain edema, antibiotics, and other symptomatic treatment measures for stroke-related complications [23]. A persistent impaired consciousness was the main reason for amantadine hydrochloride treatment [24]. Early amantadine treatment in LHI patients was defined as amantadine hydrochloride initiated within 24 h after stroke onset and continuing for at least 3 days. Amantadine hydrochloride was added at an oral dose of 100 mg twice daily. According to whether they did receive amantadine or not, the patients were divided into two groups: the amantadine group (amantadine combined with ST) and the ST group.

The study protocol was approved by the Ethics Committee of the people’s hospital of Deyang city (approval No. 2011–04-134). We obtained informed consent from all patients or their legal representative if the patient lost the capacity to give informed consent before they were enrolled, for using the patient’s data for research.

### Data collection

Demographic data, admission delay, initial stroke severity assessed by baseline National Institutes of Health Stroke Scale (NIHSS) score and Glasgow coma scale (GCS) score, baseline systolic and diastolic blood pressure, serum glucose on admission, vascular risk factors, imaging findings, stroke etiology, in-hospital treatments, and stroke-related complications during hospitalization were collected via using a standardized data collection form. Detailed methods for data collection have been described in our previous studies [19, 20]. The potential stroke etiology of LHI was classified according to the TOAST (Trial of Org 10,172 in Acute Stroke Treatment) criteria [25]. Treatments during hospitalization analyzed in the present study included intravenous thrombolysis, mechanical ventilation, and osmotic agents. Stroke-related complications, including both neurological and medical complications during hospitalization [26], were reviewed by data collectors who were not aware of the study from hospital records when the patient was discharged, which has been described in our previous study [27].

### Outcome measurement

Patients were followed up at 3 months after stroke onset by using questionnaires via telephone interview or by mail. The primary outcomes were in-hospital death, 3-month mortality, and 3-month unfavorable outcome (defined as an mRS score of 4 to 6) [22]. The secondary outcomes were stroke-related complications and adverse side effects which were recorded due to exposure to amantadine, including hypotension, livedo reticularis, seizure, hallucinations, etc.

### Statistical analyses

Statistical analyses were performed using SPSS software package (SPSS for Windows, version 22.0, Chicago, IL). Continuous variables are presented as means with standard deviations or median with range. Categorical variables are presented as frequencies with percentages. Statistical significance for intergroup differences in categorical variables were assessed by the χ^2^ test or Fisher’s exact test, while differences in continuous variables were assessed by the Student’s t-test or Mann-Whitney U test.

A propensity score matching (PSM) algorithm (logistic regression) was conducted, including baseline characteristics that are assumed to be related to the prescription of amantadine treatment, to calculate the propensity score (PS) for each patient [28]. Then the LHI patients in the amantadine group were matched with patients in the ST group by using the nearest neighbor matching approach (caliper 0.2, ratio 1:2), to minimize potential imbalances in the distribution of potential confounders between amantadine users and nonusers.

Univariate analysis was performed to test variables that may affect the outcomes of LHI patients. Multivariate logistic regression analyses were further performed to explore the association between amantadine treatment with in-hospital death, 3-month mortality, and unfavorable outcomes, by using the forced entry method adjusting for variables with *p* < 0.1 in univariate analyses. We calculated the 95% confidence intervals (CI) to describe the precision of the estimates. We also calculated 3-month survival curves via using the Kaplan-Meier method and performed a log-rank test for survival comparisons in the final matched dataset. A two-sided *p*-value < 0.05 was considered to be statistically significant.

### Data availability

The data that support the findings of this study are available from the corresponding author on reasonable request.

## Results

**Fig. 1:**
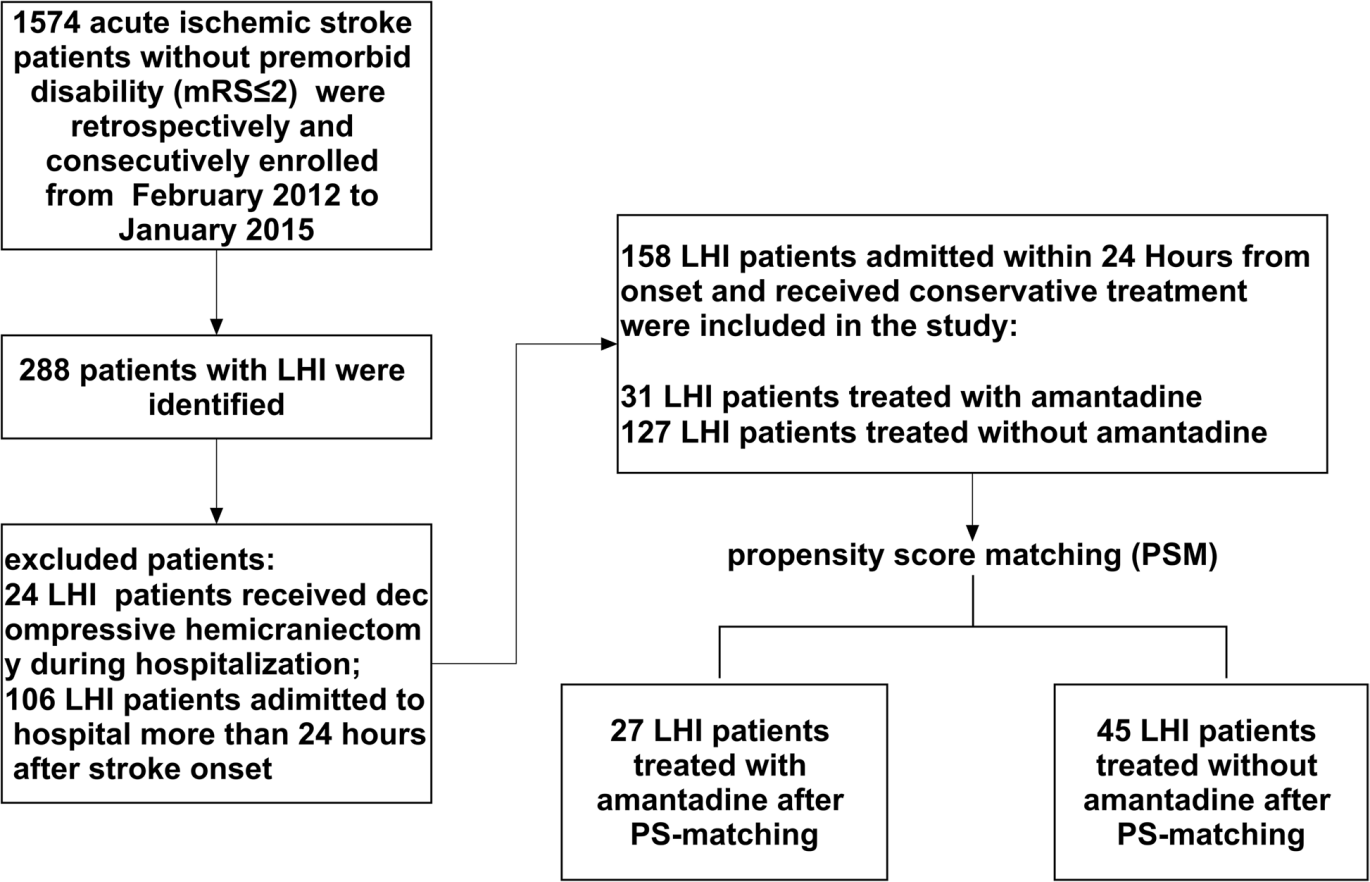
Flow diagram of included and excluded patients

During the 3-year study period, a total of 1574 AIS patients were screened. None of the patients administered intra-arterial revascularization procedures. We excluded 1416 patients. Finally, 31 LHI patients treated with amantadine combined with ST and 127 patients treated with ST were recruited to the current study. A flow diagram of included and excluded patients is provided in Fig. 1.

The baseline characteristics of LHI patients between groups are presented in Table 1. Patients treated with amantadine had a shorter prehospital delay (median: 2 vs. 10 h, *p* < 0.001), a higher baseline NIHSS score (21.71 ± 4.76 vs. 17.49 ± 5.84, *p* < 0.001) and a higher rate of dominant hemisphere involvement (67.74% vs. 45.67%, *p* = 0.028). The two groups did not differ in age, sex, baseline GCS score, baseline blood pressure and serum glucose, vascular risk factors, and stroke etiology (all *p* > 0.05).

**Table 1:** Baseline characteristics of LHI patients before PSM

	Amantadine (***n*** = 31)	ST (***n*** = 127)	***P value***
**Age (years)**			
Mean ± SD	70.68 ± 11.42	65.45 ± 15.32	0.077*
Median (range)	76 (46–87)	68 (18–94)	0.061†
Female, n (%)	17 (54.83)	69 (54.33)	0.959‡
Time from onset (hours), median (range)	2 (1–24)	10 (1–24)	< 0.001†
NIHSS score on admission	21.71 ± 4.76	17.49 ± 5.84	< 0.001*
GCS score on adimission	9.35 ± 1.72	10.17 ± 3.06	0.158*
SBP on admission (mm Hg)	150.45 ± 20.48	140.17 ± 26.96	0.056*
DBP on admission (mm Hg)	87.71 ± 14.95	82.87 ± 16.84	0.145*
Serum glucose on admission (mmol/L)	7.72 ± 4.49	7.88 ± 3.25	0.822*
**Risk factors, n (%)**			
Hypertension	12 (38.71)	66 (51.97)	0.186‡
Diabetes mellitus	6 (19.35)	27 (21.26)	0.815‡
Dyslipidemia	6 (19.35)	20 (15.75)	0.627‡
Coronary heart disease	8 (25.81)	21 (16.54)	0.232‡
Atrial fibrillation	22 (70.97)	70 (55.12)	0.109‡
Rheumatic heart disease	5 (16.13)	35 (27.56)	0.189‡
Current smoking	10 (32.26)	26 (20.47)	0.161‡
Alcohol consumption	3 (9.68)	19 (14.96)	0.446 ‡
Previous all strokes/TIA	9 (29.03)	26 (20.47)	0.304‡
Previous IS	8 (25.81)	18 (14.17)	0.117‡
Previous ICH	1 (3.23)	4 (3.15)	0.983‡
Previous TIA	0 (0)	4 (3.15)	0.716‡
**Stroke in dominant hemisphere, n (%)**	21 (67.74)	58 (45.67)	0.028‡
**TOAST classification, n (%)**			0.572‡
Large-artery atherosclerosis	5 (16.13)	32 (25.20)	
Cardio-embolism	23 (74.19)	77 (60.63)	
Other determined etiology	1 (3.23)	7 (5.51)	
Undetermined etiology	2 (6.45)	11 (8.66)	

*Abbreviations*: *PSM* Propensity score matching, *ST* Standard therapy; *SBP* Systolic blood pressure, *DBP* Diastolic blood pressure, *NIHSS* National Institutes of Health Stroke Scale, *GCS* Glasgow Coma Scale, *TIA* Transit ischemic attack, *IS* Ischemic stroke, *ICH* Intracerebral hemorrhage

*Student t test. † Mann–Whitney U test. ‡ χ2 test

After PS-matching, we identified two subgroups of 72 LHI patients that were balanced for all baseline characteristics, including 27 treated with amantadine combined with ST and 45 treated with ST. Relative multivariate imbalance in the form of the L1 measure was smaller (0.463 vs. 0.649) and no covariate had standardized mean differences of greater than 0.1 after PSM. Supplemental Fig. 1 also showed that covariate balance was massively improved in the matched dataset. As shown in Table 2, there was no significant difference in the baseline characteristics between groups in the matched dataset (all *p* > 0.05).

**Table 2:** Baseline characteristics of LHI patients after PSM

	Amantadine (***n*** = 27)	ST (***n*** = 45)	***P value***
**Age (years)**			
Mean ± SD	69.89 ± 11.56	65.73 ± 15.77	0.238*
Median (range)	76 (46–82)	69 (15–93)	0.264†
Female, n (%)	14 (51.85)	24 (53.33)	0.903‡
Time from onset (hours), median (range)	3 (1–24)	6 (1–24)	0.074†
NIHSS score on admission	21.22 ± 4.90	20.64 ± 5.65	0.661*
GCS score on adimission	9.52 ± 1.76	8.51 ± 2.85	0.103*
SBP on admission (mm Hg)	150.11 ± 26.79	145.04 ± 30.72	0.480*
DBP on admission (mm Hg)	87.78 ± 15.98	86.42 ± 18.49	0.753*
Serum glucose on admission (mmol/L)	7.15 ± 2.20	8.39 ± 3.06	0.070*
**Risk factors, n (%)**			
Hypertension	10 (37.04)	25 (55.56)	0.128‡
Diabetes mellitus	5 (16.13)	15 (33.33)	0.174‡
Dyslipidemia	6 (22.22)	8 (17.78)	0.645‡
Coronary heart disease	6 (22.22)	7 (14.89)	0.476‡
Atrial fibrillation	20 (74.07)	24 (53.33)	0.081‡
Rheumatic heart disease	4 (14.81)	11 (24.44)	0.330‡
Current smoking	9 (33.33)	10 (22.22)	0.300‡
Alcohol consumption	3 (11.11)	8 (17.78)	0.447 ‡
Previous all strokes/TIA	9 (33.33)	11 (24.44)	0.415‡
Previous IS	8 (29.63)	8 (17.78)	0.242‡
Previous ICH	1 (3.70)	3 (6.67)	0.595‡
Previous TIA	0 (0)	0 (0)	–
**Stroke in dominant hemisphere, n(%)**	17 (62.96)	27 (60.00)	0.803‡
**TOAST classification, n(%)**			0.621‡
Large-artery atherosclerosis	5 (18.52)	8 (17.78)	
Cardio-embolism	20 (74.07)	29 (64.44)	
Other determined etiology	1 (3.70)	3 (6.67)	
Undetermined etiology	1 (3.70)	5 (11.11)	

*Abbreviations*: *PSM* Propensity score matching, *ST* Standard therapy, *SBP* Systolic blood pressure, *DBP* Diastolic blood pressure, *NIHSS* National Institutes of Health Stroke Scale, *GCS* Glasgow Coma Scale, *TIA* Transit ischemic attack, *IS* Ischemic stroke, *ICH* Intracerebral hemorrhage

*Student t test. † Mann–Whitney U test. ‡ χ2 test

For the acute phase treatment of LHI, the two groups did not show differences in receiving thrombolysis, mechanical ventilation, and osmotic agents before and after PSM (all *p* > 0.05, Table 3). The mean duration of amantadine treatment was 28.71 days before PSM and 30.96 days after PSM. Outcomes of LHI patients between groups are shown in Tables 3 and 4.

**Table 3:** In-hospital treatment, stroke-related complication and outcomes of LHI patients before and after PSM

Variables	Unmatched			PS-matched		
	Amantadine (***n*** = 31)	ST (***n*** = 127)	***P*** value	Amantadine (***n*** = 27)	ST (***n*** = 45)	***P*** value
**Treatments, n (%)**						
Thrombolysis	2 (6.45)	4 (3.15)	0.735	1 (3.70)	3 (6.67)	1.000
Mechanical ventilation	1 (3.23)	6 (4.72)	1.000	1 (3.70)	5 (11.11)	0.509
Osmotic agents	29 (93.55)	110 (86.61)	0.450	26 (96.30)	42 (93.33)	1.000
**Complications, n (%)**						
Brain edema	29 (93.55)	40 (31.50)	<0.001	26 (96.30)	41 (91.11)	0.720
Hemorrhagic transformation	14 (45.16)	31 (24.41)	0.022	13 (48.15)	13 (28.89)	0.100
Seizures	4 (12.90)	11 (8.66)	0.703	4 (14.81)	7 (15.56)	1.000
Pneumonia	26 (83.87)	81 (63.78)	0.032	23 (85.19)	42 (93.33)	0.472
Gastrointestinal bleeding	6 (19.35)	22 (17.32)	0.791	6 (22.22)	6 (13.33)	0.514
Acute renal failure	7 (22.58)	15 (11.81)	0.206	6 (22.22)	8 (17.78)	0.645
**Outcomes, n (%)**						
In-hospital death	3 (9.68)	27 (21.26)	0.132	2 (7.41)	14 (31.11)	0.019
3-month mortality	9 (29.03)	51 (40.16)	0.228	7 (25.93)	25 (55.56)	0.008
3-month unfavorable outcome	19 (61.29)	74 (58.27)	0.832	16 (59.26)	32 (71.11)	0.184

*Abbreviations*: *ST* Standard therapy, *PS* Propensity score

**Table 4:** Multivariate analyses for the main outcomes of LHI patients treated with amantadine or not

Variables	Unmatched		PS-matched	
	OR	***P*** value	OR	***P*** value
**Death in-hospital**^a^	0.143 (0.034–0.605)	0.008	0.113 (0.020–0.635)	0.013
**3-month mortality**^b^	0.214 (0.077–0.598)	0.003	0.176 (0.053–0.586)	0.005
**3-month unfavorable outcomes**^c^	0.501 (0.192–1.309)	0.158	0.344 (0.105–1.126)	0.078

*Abbreviations*: *PS* Propensity score

Figures in parentheses are 95% confidence intervals (CI)

^a^ Adjusted for age, sex, baseline NIHSS score and serum glucose on admission

^b^ Adjusted for age, sex, baseline NIHSS score

^c^ Adjusted for age, sex, baseline NIHSS score and vascular risk factors (history of hypertension)

### The primary outcomes

#### In-hospital death

Before PSM, LHI patients treated with amantadine did not show a significantly lower rate of in-hospital death (9.68% vs. 21.26%, *p* = 0.132). However, amantadine treatment significantly reduced the risk of in-hospital death after PSM (7.41% vs. 31.11%, *p* = 0.019).

After adjusting for age, sex, baseline NIHSS score and serum glucose on admission, the multivariate analysis yielded a significant decrease in death in-hospital (OR 0.143, 95% CI 0.034 to 0.605 before PSM and OR 0.113, 95% CI 0.020 to 0.635 after PSM, respectively, both *p* < 0.05).

#### 3-month mortality

**Fig. 2:**
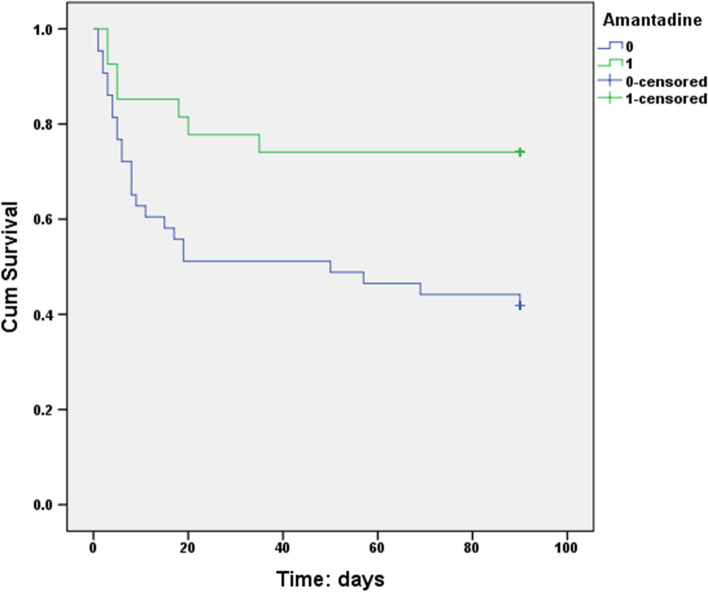
3-month survival curves for LHI patients treated with amantadine or not (after PSM)

At 3 months, 1.2% (2/158) patients were lost to follow-up, both were in the ST group. Before PSM, LHI patients treated with amantadine did not show a significantly lower rate of 3-month mortality (29.03% vs. 40.16%, *p* = 0.228). However, amantadine treatment reduced the risk of 3-month mortality after PSM (25.93% vs. 55.56%, *p* = 0.008). 3-month survival was estimated by the Kaplan-Meier method and identified that amantadine was associated with a significantly higher 3-month survival rate (*p* = 0.020, log-rank test, Fig. 2).

After adjusting for age, sex, baseline NIHSS score, the multivariate analysis yielded a significant decrease in 3-month mortality (OR 0.214, 95% CI 0.077 to 0.598 before PSM and OR 0.176, 95% CI 0.053 to 0.586 after PSM, respectively, both *p* < 0.05).

#### 3-month unfavorable outcomes

Amantadine treatment was not associated with a significantly lower rate of 3-month unfavorable outcomes before and after PSM, no matter in univariate or multivariate analyses (all *p* > 0.05). Nevertheless, 11 (40.7%) patients in the amantadine group and 11 (25.6%)in the ST group had an mRS score of ≤3 after PSM, which suggested a nonsignificant trend towards 3-month favorable outcomes in those LHI patients treated with amantadine (Supplemental Fig. 2).

### The secondary outcome

Before PSM, the amantadine group had a significantly higher rate of brain edema (93.55% vs. 31.50%, *p* < 0.001), hemorrhagic transformation (45.16% vs. 24.41%, *p*=0.022) and pneumonia (83.87% vs. 63.78%, *p* = 0.009). However, the two groups did not differ in all stroke-related complications after PSM (all *p* > 0.05). It is worth noting that the amantadine group did not have a higher rate of seizures before and after PSM.

Amantadine hydrochloride was well tolerated at a dosage of 200 mg/day throughout the study. There were no serious adverse side effects recorded due to exposure to amantadine. No patients experienced discontinuation of medication or change in dosage throughout the study because of side effects.

## Discussion

The present study determined whether amantadine treatment is associated with better clinical outcomes in conservatively treated LHI patients. To the best of our knowledge, this is the first study to investigate the treatment effect of amantadine in patients with severe stroke. Although it was not a randomized study, and there might be some subtle differences in clinical presentation which would have influence on the decisions of treating clinicians to prescribe amantadine or not. However, in the present study, the amantadine group had a higher baseline NIHSS score (21.71 ± 4.76 vs. 17.49 ± 5.84) and higher rate of dominant hemisphere involvement (67.74% vs. 45.67%) than the ST group before PSM. The amantadine group also had a higher rate of brain edema (93.55% vs. 31.50%) and hemorrhagic transformation (45.16% vs. 24.41%). Neither supports that treating clinicians tend to prescribe amantadine in the subjects most likely to survive. Our results suggested that early amantadine treatment was associated with significantly lower rates of in-hospital and 3-month death and nonsignificant trend of favorable outcomes in conservatively treated LHI patients, after adjusting for age, baseline NIHSS score, and other confounders which might influence the decisions to prescribe amantadine, or in the final baseline characteristics-matched dataset. In our study, a total of 30 patients died during hospitalization and 60 patients died at 3-month follow-up. The most common cause of 3-month death was brain herniation in both groups (6 patients in the amantadine group and 26 patients in the ST group), followed by pneumonia (3 patients in the amantadine group and 13 patients in the ST group). For financial concerns, religion, and values, some critically ill patients were given up treatment by their family members and died within a few days after discharge, especially those patients from rural areas. In the present study, 6 patients in the amantadine group and 24 patients in the ST group died after discharge, however, among those patients, 5 (83.33%) in the amantadine group and 10 (41.47%) in the ST group had withdrawal of care. Thus, our results could not be explained by that amantadine resulted in more wakefulness then perhaps families would have withdrawn care less often making it appear that amantadine resulted in lower mortality. Meanwhile, most of the survivors with severe disabilities were taken home directly or opted for hospice care, rather than seeking further speech therapy and physiotherapy, which could explain why the follow-up data from LHI patients recovering from such a devastating condition are so close to their discharge [27].

Stroke is the second most common cause of death worldwide and the leading cause of death in China [29]. Ischemic stroke, which accounts for approximately 80% of all strokes, is a serious disease with a complex pathophysiology. Various agents that may interfere with each step of the ischemic cascade can, in theory, be developed as a candidate for stroke treatment [30]. Two major strategies have been developed to treat ischemic stroke: recanalization (including thrombolysis and mechanical thrombectomy) and neuroprotection [31]. During the last three decades, thousands of neuroprotective agents have been tested in animal models with reports of experimental efficacy, and nearly 200 neuroprotection clinical trials are ongoing or have been completed, but none has been proven effective in clinical trials [32]. Despite the failure of most neuroprotective drugs, neuroprotection has never been abandoned especially with the help of improved preclinical testing and clinical trial design [33, 34].

LHI, which usually results from a failure in successful reperfusion of occlusion of the internal carotid artery or proximal MCA, is a devastating disease with high mortality rate [1–3]. Although DHC has been proven to benefit LHI patients with malignant course [5], only highly selected patients younger than 60 years of age would be eligible for DHC [6]. Until recently, no pharmacological strategies have been proven effective by clinical trials [4]. Despite the lack of evidence, many neuropharmacological therapies are still used off-label in clinical practice due to the lack of effective treatment in conservatively treated LHI patients.

In the present study, we found that early amantadine treatment was associated with a significantly lower death rate and nonsignificant trend of favorable outcomes in conservatively treated LHI patients. These results are similar to a clinical-experimental study that was carried out in patients with AIS in Russian and reported that amantadine sulfate (PK-Merz) exhibited significant restoration of consciousness and better regress of neurological deficit in the first hours of AIS [18]. There are two main reasons for the effects of amantadine. First, amantadine has profound NMDA receptor antagonist effects, which may contribute to its neuroprotective effects when administered in the first several hours or days after brain injury [16]. It is theorized that it can block the activation of glutamate receptors and other NMDA channels to inhibit the elevation of intracellular Ca^2+^ levels. Excess of intracellular Ca^2+^ plays a unique role in the ischemic pathophysiology by activation of a variety of Ca^2+^ dependent enzymes, leading to irreversible mitochondrial damage, inflammation, cytotoxic edema, necrotic and programmed cell death [35, 36]. Second, amantadine may promote dopaminergic activity by facilitating presynaptic release and blocking postsynaptic reuptake, which is related to the enhancement of endogenous recovery mechanisms. The favorable functional consequences of amantadine may reflect the strengthening of neurotransmission in the dopamine-dependent nigrostriatal, mesolimbic, and frontostriatal circuits that are responsible for mediating arousal, drive, attentional, and cognitive functions [37, 38]. A systematic review concluded that improvement in arousal and cognitive function had been observed in patients with TBI when amantadine was initiated 3 days to 5 months after brain injury [39]. Two case studies using positron emission tomography to evaluate the effects of amantadine on chronic TBI showed that amantadine treatment would result in arousal and executive function improvement and a significant increase in prefrontal cortical metabolism and a nonsignificant increase in striatal D2 dopamine-receptor availability [40, 41]. Results of the above studies support the role of amantadine in enhancing neurological rehabilitation through the dopaminergic system independent of its neuroprotective effects. In a multicenter trial, amantadine with a treatment duration of 4 weeks has successfully been demonstrated effective and safe in accelerating the pace of functional recovery in patients with severe TBI, and the investigator of the trial indicated that the response of amantadine is drug-dependent [17]. In our study, the mean duration of amantadine treatment was 28.71 days before PSM and 30.96 days after PSM, however, we did not find a significant effect in 3-month functional outcome. Since amantadine has the dual effect of neuroprotection and recovery enhancement, it is reasonable to choose a treatment duration of 4 weeks or longer in future research.

The seizure is a side effect of the biggest concern to clinicians when treating LHI patients with amantadine. However, poststroke seizures are not frequent in LHI patients with an incidence rate of 7.0% in our previous study [27]. In our study, LHI patients treated with amantadine did not suffer a higher rate of seizures before and after PSM. Amantadine’s effect on the seizure threshold is dose-dependent. It has been demonstrated that lower doses of amantadine may elevate the seizure threshold, while high doses (greater than 400 mg/day in adults) may induce seizures [42]. Similar dosages of amantadine to our study even may be an effective alternative treatment for some types of seizures [43, 44], which most likely results from its NMDA receptor antagonist effects [45].

It’s worth noting that so few LHI patients received intravenous thrombolysis and mechanical ventilation in the present study. This could be explained by that our study was a retrospective, Chinese hospital-based study enrolling LHI patients admitted from February 2012 to January 2015. In that period, the rate of thrombolysis in China is relatively low, and early CT hypodensity sign and severe stroke were considered as contraindications or relative contraindications to intravenous thrombolysis, so clinicians were less positive in performing intravenous thrombolysis in patients with anterior circulation large vessel occlusion. In the area where our hospital located, many families hesitate to accept invasive treatments such as mechanical ventilation due to financial concerns, religion, and other values. In our previous published study [27], which included LHI patients from October 2011 to September 2014, only 2.7% cases received thrombolysis, and only 11.7% cases were treated with mechanical ventilation. In the current study, we excluded LHI patients who had received DHC during hospitalization (who were more likely to receive mechanical ventilation), so the rate of cases receiving mechanical ventilation was even lower than that reported in our previous study. As a result, the effect of amantadine needs to be confirmed in LHI patients who had received reperfusion therapy and mechanical ventilation in further study.

### Limitations

The present study has several limitations, so our results should be interpreted with caution. First, it was a single tertiary hospital-based study conducted in China, so the results may not represent the whole population. Some patients with severe stroke might not be hospitalized, especially those who died before hospitalization, so we could not exclude inclusion bias. Second, it was a retrospective study, so we could not control the duration of amantadine and other combination therapy. Although it was not a randomized study, and there might be some subtle differences in the clinical presentation which would influence the decisions of treating clinicians to prescribe amantadine or not, we performed a PSM analysis to minimize potential imbalances between groups on baseline characteristics that are assumed to be related to the prescription of amantadine treatment. Third, the sample size of the amantadine group was relatively small and reduced the likelihood of finding significant effects. Fourth, although we did not calculate and compare the infarct volume that is critical to the outcomes of LHI patients, PSM analysis was performed to make the baseline NIHSS score balanced between groups in the current study. Fifth, we only conducted follow-up at a single time point of 3 months. Meanwhile, follow-up in our study was conducted via telephone interview or mailed questionnaire instead of a clinical visit, which may increase the risk of reporting bias. Finally, we did not use continuous electroencephalography routinely to detect seizures. However, a high incidence of amantadine-induced subclinical seizures would be expected to lead to more unfavorable outcomes.

## Conclusion

The results of our study provide initial evidence that early amantadine treatment was associated with a significantly lower death rate and nonsignificant trend of favorable outcomes in conservatively treated LHI patients. Considering the limitations of observational study, randomized controlled trials with a large sample size may help provide a clearer picture of the utility of amantadine in LHI patients.

## Supplementary Information

**Additional file 1.**

## Data Availability

The data that support the findings of this study are available from the corresponding author on reasonable request.
