# Supplementary material for: Early amantadine treatment reduces the risk of death in patients with large hemisphere infarctions:a Chinese hospital-based study
Source: BMC Neurol. 2021 Oct 28;21:419. doi: 10.1186/s12883-021-02444-w (PMC8554877; doi:10.1186/s12883-021-02444-w)

**Early amantadine treatment reduces the risk of death in patients with large hemisphere infarctions**

Jie Li**^ab^**, MD; Ping Zhang**^a^**, MD; Yingying Liu^a^, MD; Simiao Wu **^b^**, MD, PhD; Xingyang Yi**^a^**, MD; Shihong Zhang^b^, MD, PhD; Chun Wang**^a*^**; Ming Liu **^b*^**, MD,PhD

^a^ Department of Neurology, People’s Hospital of Deyang City, Deyang, PR China.

^b^ Center of Cerebrovascular Diseases, Department of Neurology, West China Hospital, Sichuan University, Chengdu, PR China.

***Co-corresponding author**: Chun Wang, Department of Neurology, People’s Hospital of Deyang City, No.173, North Taishan Road, Deyang 618000, Sichuan Province, PR China. E-mail: dysrmyysjnkwc@163.com; Ming Liu, Center of Cerebrovascular Diseases, Department of Neurology, West China Hospital, Sichuan University, No. 37 GuoXue Xiang, Chengdu, Sichuan Province 610041, PR China. E-mail: [wyplmh@hotmail.com](mailto:wyplmh@hotmail.com).

**Supplemental figure 1. Histograms with overlaid kernel density estimates of standardized differences before and after matching.**


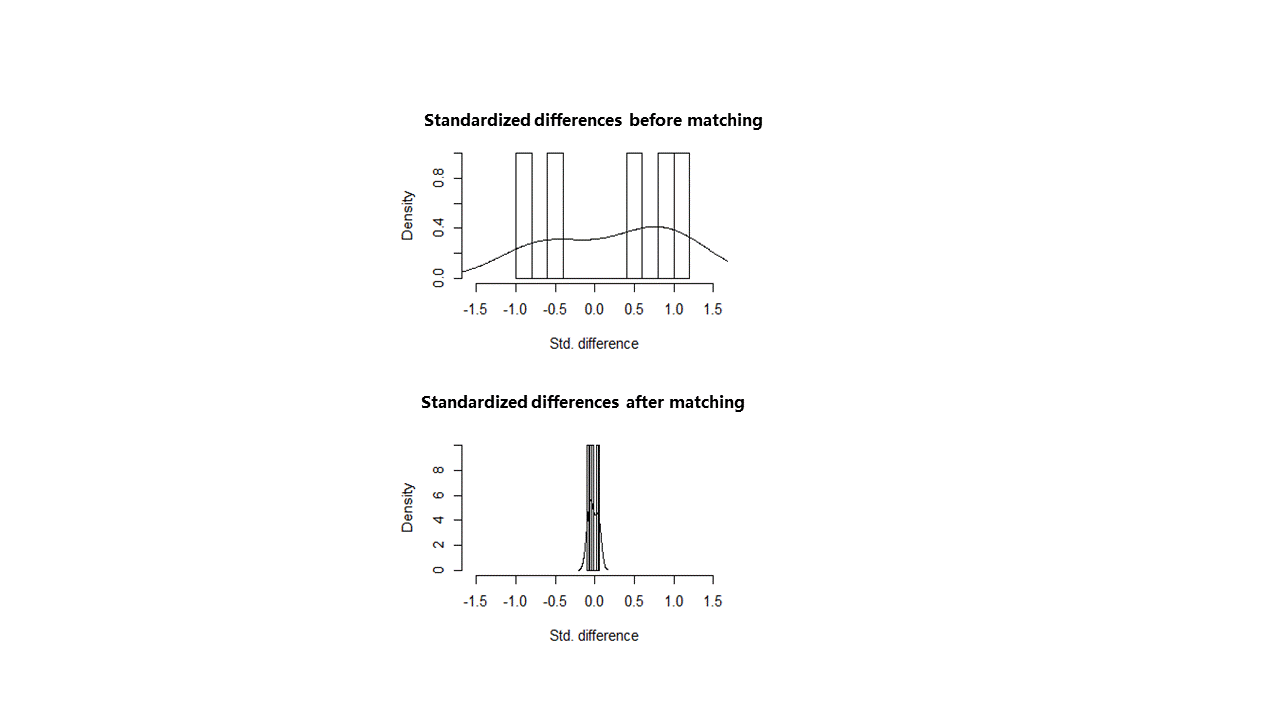


**Supplemental figure 2. Distributions of the mRS score at 3 months for LHI patients treated with amantadine or not (after PSM).**


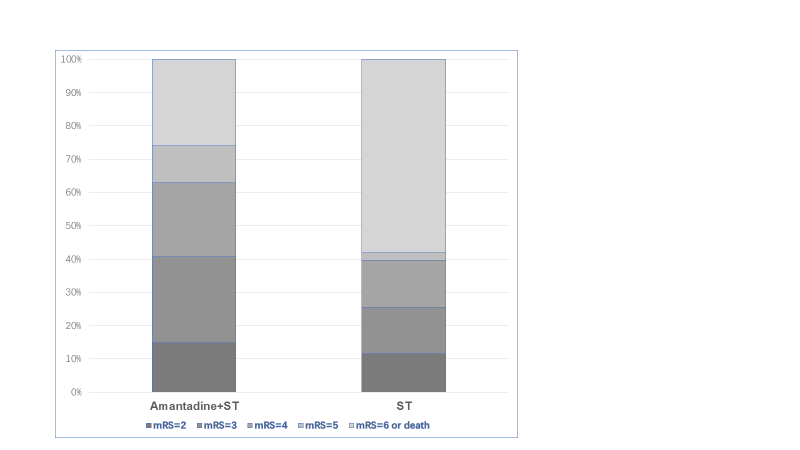

Supplement: Supplementary file 1 — Additional file 1. [file 12883_2021_2444_MOESM1_ESM.docx]
